# Mental Rest

**Published:** 1879-11

**Authors:** 


No man can study advantageously all the time ; there must be some relaxation, or the brain will become disorganized, or otherwise hopelessly diseased. No man ought to study hard on one subject more than four hours a day, and give ten hours to purposes of dressing, sleeping and eating, leaving ten hours for mental recreation, that is, mental rest, which is done in two ways: first, engaging the mind in thinking about something else ; that is, putting other organs of the brain to work; second, engaging in muscular motion, which does good in two ways; it works out of the system the waste particles made by hard thought, and thus purifies the blood, fitting it for building up the brain, repairing its wastes, making it ready for new work. But muscular motion does good in another way ; the mind is diverted to it, and is thus rested from the main study ; hence the true policy of hard students is not to sit, or loll, or lounge about, but to be doing something with the hands or feet which is of sufficient interest to engage the mental notice, and, if pleasurably engaged, so much the better, even by fifty or a hundred fold, hence the three employments, or side works, of the three great men named were really mental rests, recreations, whether it was Trousseau playing watchman in his hay-loft, or Newton feeling his pipe with his sweetheart's finger, or the editor of Hall's Journal of Health! clearing the flags of snow in shirt and pants at daylight, of a December morning, for an hour or two We rather think that our mode of resting the mind was the most utilitarian, plilosophical, humane, and least wickedly risky of the three.

It will be useful to remark here that we do not like to use our eyes in reading or writing until breakfast has been eaten, because they are stronger all the day afterwards; nor do we use them in that way after twilight; this has been our uniform habit, with the result, as we think, that we were able to do without the use of glasses for eight or ten years after our old school-mates of the same age had been using them, and we are writing now without any glasses whatever. Albert Barnes, the eminent commentator, rose habitually at four o'clock for study, and soon had to abandon all study, go abroad, and, after years of lost time, prematurely laid on the shelf from diseased eyes.

Literary and professional men, in consulting us, often inquire, with great earnestness, how can we take exercise in a large city, or town or village—"there is nothing that we can do." In the first place, eat about one-half less every day, and you will at once require but half the amount of exercise; now for the remainder. Have you a family? If you have not, you need not take any exercise, and you can eat, and stuff, and guzzle all day, for you are of no account, and the sooner you die off and make room for a better man, the better for society at large. But taking it for granted that you have a family, like other respectable men, and live in a brown stone house on Murray Hill, New York, there are a multitude of very useful things you can do every day to the comfort of your family, the benefit of your health, the improvement of your digestion, the soundness of your sleep, the vigor of your thought, and the benignity of your disposition, get up at five o'clock winter and summer, go down into the cellar, riddle out all the cinders of the day before from range, furnace and grates, sprinkle them with water, and put them in the furnace; if you are hardy and systematic you can do all this in half an hour, and save about half a dollar besides, if you have a good-sized family; next help your servant girls, giving them a chance to sleep a little longer, by kindling a few of the fires, and, if you are handy, you will save a good deal of paper and wood-kindling every day.

Literary and professional men maintain an idle theory when they consider every moment lost which is not employed in reading, writing, or investigation; it is loss of time in the long run which should alarm the individual; it is the curtailment of human life for ten, twenty, and even thirty years, which should startle the mind and lead to a wiser way of life. Whoever indulges in brain-work over four hours a day habitually, does in proportion shorten his life, or at least shortens the term of his usefulness; this is a great general rule, to which there may be some exceptions; but let the reader take it for granted that he is not one of those exceptions; on the contrary, whatever of time spent in muscular activities, beyond the four hours of brain work, adds that much to the probabilities of a longer life, and a life, too, of greater efficiency.

